# Clinical Evaluation of a Synthetic Hybrid-Scale Matrix in the Treatment of Lower Extremity Surgical Wounds

**DOI:** 10.7759/cureus.27452

**Published:** 2022-07-29

**Authors:** Daniel Tucker

**Affiliations:** 1 Podiatry - Foot and Ankle Surgery, Vascular and Vein Institute/Limb Preservation Center of the South, Germantown, USA

**Keywords:** wound healing, hybrid-scale fiber matrix, synthetic, operative wounds, lower extremity

## Abstract

Lower extremity operative wounds can be difficult to treat and are associated with social challenges. New treatment options are needed to mitigate the clinical and social challenges and potentially lower the treatment cost and time. A synthetic hybrid-scale fiber matrix, which has an architecture similar to native tissue, a tailored resorption rate, and excellent durability and handling characteristics, is gaining popularity due to its effectiveness in treating different kinds of wounds. In this retrospective study, nine patients with a total of nine lower extremity wounds and multiple comorbidities were included. The hybrid-scale fiber matrix was cut to wound size, fenestrated as deemed appropriate, and secured to the wound bed using staples or sutures. Wound healing was monitored at subsequent visits, and the synthetic fiber matrix was re-applied as needed. Healing was considered complete when 100% epithelialization and closure of the wound site occurred. Overall, seven out of nine wounds exhibited complete healing and wound closure. For fully healed wounds, the average time to heal was 135.6 days, and good scar quality was observed. This retrospective case series demonstrates the versatility of synthetic hybrid-scale fiber matrices as part of an effective treatment regimen for difficult-to-treat lower extremity operative wounds.

## Introduction

Nearly 15% of Medicare beneficiaries are impacted by wounds, and the estimated cost associated with wound care is $28 billion [1]. This cost can increase significantly, from $31.7 billion to $96.8 billion, if wounds are included as a part of a secondary diagnosis, and this estimate does not include individuals not in the Medicare program [1]. The magnitude of wounds as a healthcare problem is on the rise, and continued attention to the education, care, and research of wounds is required [2]. Rising healthcare costs, an aging population, diabetes, obesity, stress, and malnutrition are a few factors that contribute to the ever-increasing clinical, social, and economic challenges of treating wounds [2]. Given these challenges, it is imperative to develop treatments that can lead to positive outcomes in terms of rapid healing and complete wound closure, along with the minimal risk of complications.

Recently, a synthetic hybrid-scale fiber matrix has gained popularity due to its promising results, when used to treat various kinds of wounds [3-5]. Biologic materials or substrates are commonly used to treat wounds; however, they carry the risk of disease transmission, inflammatory response, and infection [3]. Fully synthetic materials can be considered as a solution to address the issues with biological materials. Specifically, the synthetic hybrid-scale fiber matrix is an optimal solution due to its biocompatibility (composed of FDA-approved synthetic polymers, polyglactin 910, and polydioxanone) and fibrous structure with high porosity similar to the native extracellular matrix [3]. Successful clinical applications of this synthetic matrix have been shown in lower extremity wounds such as chronic neuropathic foot ulcers as well as in trauma and acute care wounds [5-12].

This retrospective case series evaluates the use of the synthetic hybrid-scale fiber matrix for the treatment of lower extremity wounds resulting from various types of amputations, such as transmetatarsal, metatarsal/partial ray, and Lisfranc amputations. This study was based on the hypothesis that the synthetic hybrid-scale fiber matrix would contribute to effective wound healing and closure, with no or minimal complications or side effects.

This article was previously presented as a poster at the 2022 Symposium on Advanced Wound Care (SAWC) Spring Conference from April 7 to 11, 2022.

## Materials and methods

A retrospective case series was conducted to assess the use of a synthetic hybrid-scale fiber matrix (Restrata®, Acera Surgical, St. Louis, MO) for the treatment of lower extremity wounds in conjunction with other advanced treatment modalities. The study was exempt from institutional review board approval due to the retrospective nature and absence of patient identifying information. A total of nine patients treated with an initial application of the synthetic material between September 2020 and March 2021 were included.

The wound size was measured. The hybrid-scale fiber matrix was cut to wound size, fenestrated as deemed appropriate, and secured to the wound with staples or sutures at the clinician’s discretion. All the wounds were treated with wound dressings (Adaptic, 3M, Maplewood, MN) and negative pressure wound therapy (NPWT) after the synthetic fiber matrix was applied. At the clinician’s discretion, five of the nine wounds were also treated with amniotic tissue, one of the nine wounds was treated with skin grafting, two of the nine wounds were treated with both amniotic tissue and skin grafting, and one of the nine wounds did not receive either treatment.

Wound healing was monitored at subsequent visits, and the synthetic fiber matrix was re-applied as needed. Healing was considered complete when 100% epithelialization and closure of the wound site occurred. The number of synthetic matrix applications, scar quality, primary healing time in days, any infections, and any complications as well as types of complications were recorded.

## Results

A total of nine patients with a total of nine lower extremity wounds were included in this retrospective case series. Table 1 provides the details of all cases included in this study, and Table 2 provides the summary of the results obtained. Wound types included four transmetatarsal amputations, one Lisfranc amputation, and four metatarsal/partial ray amputations. The average patient age was 59 years old, and the average initial wound size was 49.7 cm^2^. Patients had various comorbidities, including diabetes mellitus type 2, hypertension, smoking, obesity, diabetes mellitus neuropathy, chronic kidney disease, peripheral artery disease, atrial fibrillation, gastroparesis, congestive heart failure, and coronary artery disease.

**Table 1:** Individual patient case details. NPWT: negative pressure wound therapy; SG: skin graft; AT: amniotic tissue.

	Case #1	Case #2	Case #3	Case #4	Case #5	Case #6	Case #7	Case #8	Case #9
Age (at time of surgery, years)	57	56	64	63	52	56	55	68	66
Wound size (length, width, depth, cm)	15 x 6 x 2	9 x 5 x 2	9 x 4 x 1	7.5 x 6 x 1; 4 x 5 x 1	6 x 4.5 x 1	4 x 4 x 1	12.5 x 10 x 1	5 x 5 x 1	9 x 2 x 1
Number of synthetic matrix pieces used	1	1	1	1	1	1	1	1	1
Postoperative interventions	Wound care, NPWT, AT	Wound care, NPWT, AT	Wound care, NPWT, AT	Wound care, NPWT, SG	Wound care, NPWT, AT	Wound care, NPWT	Wound care, NPWT, SG, AT	Wound care, NPWT, SG, AT	Wound care, NPWT, AT
Primary healing	Yes	No	Yes	Yes	Yes	No	Yes	Yes	Yes
Time to heal (days)	215	-	175	50	122	-	144	144	99
Scar quality	Good	-	Good	Good	Good	-	Good	Good	Good
Major wound complications	No	Yes	No	No	No	Yes	No	No	No
Type of complications	-	Dehiscence	-	-	-	Infection	-	-	-

**Table 2:** Summary of patient demographics and wound healing characteristics. Data reported where available.

Demographic and wound healing data collected	Total wounds (n = 9 patients)
		Patient age	Mean: 59.0 (range: 52-68) years
Male/female	5/4
Wound types	Transmetatarsal amputation (4 wounds)
	Lisfranc amputation (1 wound)
	Metatarsal/partial ray amputation (4 wounds)
Presence of diabetes mellitus type 2	8 out of 9 patients
Initial wound surface area, mean ± SD (cm^2^)	49.7 ± 35
Primary healing	7 out of 9
Time to heal, mean ± SD (days)	135.6 ± 49.1
Scar quality upon healing	Good (all wounds)
Number of synthetic hybrid-scale fiber matrix applications per case	1
Complications	2, unrelated to the synthetic hybrid-scale fiber matrix

**Figure 1:**
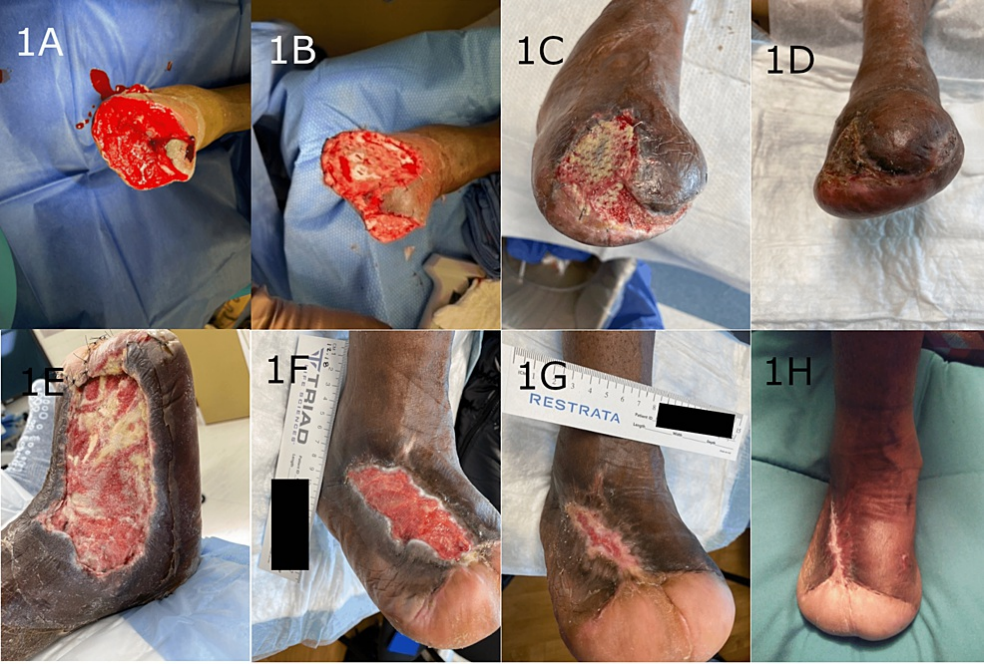
Examples of two transmetatarsal amputation wounds demonstrating progressive healing and complete closure following treatment with the synthetic hybrid-scale fiber matrix. A: Transmetatarsal amputation with initial wound size measuring 7.5 x 6 cm and 4 x 5 cm. B: Initial application of the synthetic hybrid-scale fiber matrix in the operating room. C: Second visit with initial application of the synthetic hybrid-scale fiber matrix resorbing into the wound bed. Wound demonstrating continued healing. D: Complete healing of the transmetatarsal wound seven weeks after the initial application of the synthetic hybrid-scale fiber matrix. E: Initial presentation of the transmetatarsal amputation wound prior to application of the synthetic hybrid-scale fiber matrix measuring 12.5 x 10 cm. F: Transmetatarsal amputation wound 14 weeks after the initial application of the synthetic hybrid-scale fiber matrix. G: Continued healing of the transmetatarsal amputation wound 19 weeks after initial application. H: Complete healing of the transmetatarsal amputation wound 20 weeks after initial application.

**Figure 2:**
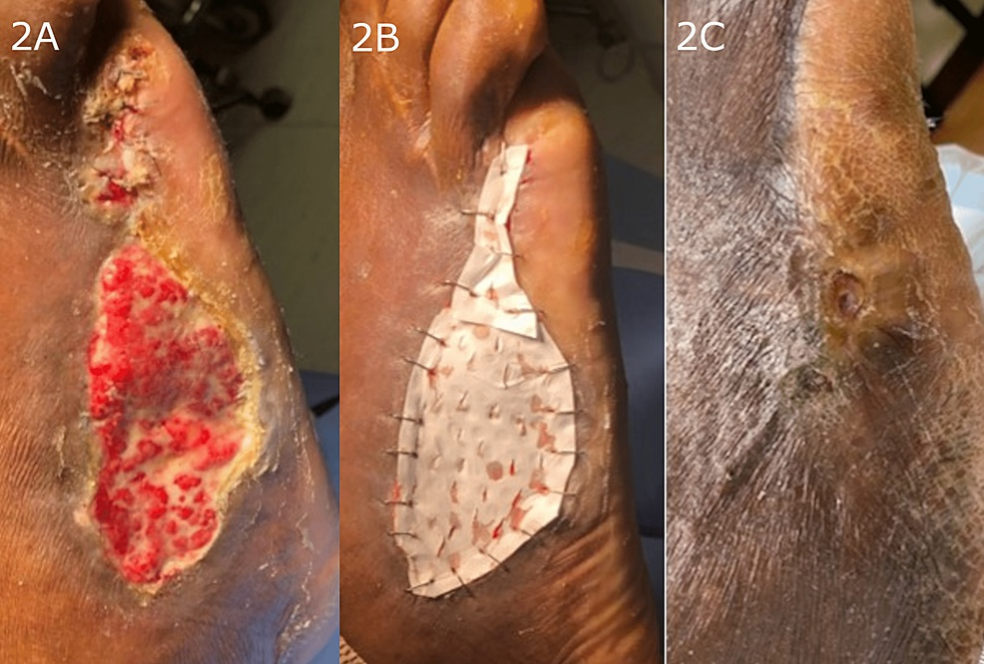
Example of metatarsal/partial ray amputation wound demonstrating progressive healing and complete closure following treatment with the hybrid-scale fiber matrix. A: Initial presentation of the metatarsal/partial ray amputation wound measuring 9 x 4 cm. B: Initial application of the synthetic hybrid-scale fiber matrix. C: Complete closure of the metatarsal/partial ray amputation wound.

Overall, seven out of nine wounds exhibited complete healing and wound closure (Figures 1, 2). The average time to heal, in cases of fully healed wounds, was 135.6 days, and good scar quality was observed. The two wounds that did not exhibit complete healing were due to complications unrelated to the synthetic matrix. For one of the wounds that did not heal (Lisfranc amputation), wound dehiscence was observed that was unrelated to the synthetic hybrid-scale fiber matrix. The second wound that did not heal (a metatarsal/partial ray amputation) became infected and re-opened due to patient non-compliance; follow-up treatment consisted of re-operation and additional application of the synthetic hybrid-scale fiber matrix. All wounds received one application of the hybrid-scale fiber matrix during the course of treatment.

## Discussion

In the United States, approximately 150,000 patients per year undergo lower extremity amputations, which can be caused by diabetes mellitus, peripheral vascular disease, malignancy, and trauma [13,14]. This retrospective study evaluated the use of a synthetic hybrid-scale fiber matrix in the treatment of complex operative wounds. Common materials for treating wounds can either be biologic in nature, synthetic, or a combination of both. For biologic materials, there is a risk of disease transmission, inflammatory response, or infection, whereas current synthetic materials suffer from a lack of native tissue architecture and suitable biocompatibility profiles [3]. In contrast, the recently developed synthetic hybrid-scale fiber matrix addresses these concerns. The synthetic matrix has an architecture similar to the native extracellular matrix [3]. The matrix itself is made from two resorbable polymers. These polymer fibers have an average diameter of <2000 nm, closely resembling the size and structure of the natural human extracellular matrix [3]. This engineered design encourages cellular ingrowth, differentiation, and neovascularization [3]. The synthetic hybrid-scale fiber matrix has been applied to various types of wounds with positive outcomes [5-8,10-12]. Additionally, previous work has demonstrated economic benefits of the product compared with biologics, including cost savings associated with less operating room time, less operative time, lower total cost of care, and indirect cost savings associated with advantages resulting from the handling, storage, and preparation benefits [7].

Primary healing was observed in seven out of nine wounds treated with the synthetic matrix, a comparable healing rate to previously published results that reported that primary healing of below-knee amputations ranged from 30-92% [15]. The two wounds that did not heal had complications (dehiscence and infection) unrelated to the synthetic hybrid-scale fiber matrix. These complications are not uncommon and have been reported in other studies [16,17]. In addition, significant healing was obtained following one application of the synthetic hybrid-scale fiber matrix in the majority of the patients, similar to results achieved by Barton et al., even though the wound size was much larger in this study compared to Barton et al.'s study (49.7 ± 35 cm^2^ vs. 18.7 ± 18.8 cm^2^) [6].

Multiple therapies were used during the course of treatment based on clinician discretion, including skin grafting, amniotic tissue, and NPWT. The healing time in this study ranged from 7.1 weeks to 30.7 weeks, with seven out of nine wound closures reported by the end of the study duration. These results highlight the use of the synthetic matrix in concert with other advanced therapies, demonstrating the matrix’s versatility and its ability to be used together with other therapies to achieve wound closure, especially for difficult-to-treat wounds and in the presence of multiple comorbidities. The use of the synthetic hybrid-scale fiber matrix in conjunction with other advanced treatment modalities has its benefit. The matrix can be used either independently or with NPWT to re-granulate wounds in preparation for a skin graft [11]. In a previous retrospective case series of pressure ulcers, the ulcers were treated with the synthetic hybrid-scale fiber prior to flap reconstruction [11]. It was observed that no ulcers experienced wound dehiscence, despite this occurring in 31% of flap reconstructions [11]. Regulski et al. have also shown that the synthetic hybrid-scale fiber matrix is effective in healing lower extremity wounds when used alone, with 85% wound closure obtained at 12 weeks [5]. The longer healing time and use of multiple modalities in the current study as compared to Regulski et al.'s study may be attributed to wounds that were more difficult to heal in the present study, as suggested by the larger initial average wound size (3.35 ± 4.74 cm^2^ vs. 49.7 ± 35 cm^2^) and presence of multiple comorbidities [5]. The current study further depicts the versatility of the synthetic hybrid-scale fiber matrix. The positive outcomes seen in the present study may be attributed to the combined effect of the therapies leading to improved wound healing for these challenging operative wounds.

This retrospective case series demonstrated positive wound healing outcomes (seven out of nine wound closures). However, there are limitations associated with this. Multiple treatment modalities were used in conjunction with the synthetic hybrid-scale fiber matrix. While this demonstrates the versatility of use, the number of variables this introduces can make it difficult to draw conclusions relating directly to the use of the synthetic hybrid-scale fiber matrix. Only a small number of patients were included, and a control group was not established. In addition, the present case series was retrospective in nature and therefore subject to selection and recall bias [18]. This was also completed at a single site with one healthcare professional. Future work is warranted to further elucidate the benefits of the synthetic matrix on the wound healing process and a randomized controlled trial should be considered to further investigate the results seen in this limited retrospective case series.

## Conclusions

Wounds resulting from lower extremity amputations can be difficult to treat, and other potential complications such as infection, tissue necrosis, pain, and dehiscence can occur. Complications can result in extended hospital stays, increased costs, and increased rates of morbidity and mortality. To address these issues, wound treatments that can help rapidly re-granulate and re-epithelialize the wound and promote healing are needed. This would help to achieve short healing periods, thus resulting in quick patient recovery, rapid mobility gain, and improved quality of life, as well as lower the burden on the healthcare system.

This retrospective case series demonstrates the versatility of the synthetic hybrid-scale fiber matrix in conjunction with other advanced treatment modalities to achieve positive clinical outcomes. The results reported in this study suggest that this matrix is a viable and safe treatment option for hard-to-treat wounds and can be used in concert with other treatment options to promote healing and wound closure.
